# Accessing the stapedius muscle via novel surgical retrofacial approach during cochlear implantation surgery: Intraoperative results on feasibility and safety

**DOI:** 10.1371/journal.pone.0272943

**Published:** 2022-08-11

**Authors:** Orlando Guntinas-Lichius, Dirk Arnold, Gerd Fabian Volk, Daniela Korth, Rene Aschenbach, Johann-Martin Hempel, Fritz Schneider, Thore Schade-Mann, Philipp Gamerdinger, Anke Tropitzsch, Hubert Löwenheim

**Affiliations:** 1 Department of Otorhinolaryngology, Jena University Hospital, Jena, Germany; 2 Department of Radiology, Jena University Hospital, Jena, Germany; 3 Department of Neuroradiology, University of Tübingen Medical Center, Tübingen, Germany; 4 Department of Otolaryngology-Head & Neck Surgery, Hearing Research Center, University of Tübingen Medical Center, Tübingen, Germany; University of Miami School of Medicine, UNITED STATES

## Abstract

Human stapedius muscle (SM) can be directly and safely accessed via retrofacial approach, opening new approaches to directly measure the electrically evoked stapedius reflex threshold (eSRT). The measurement of the SM activity via direct surgical access represents a potential tool for objective eSRT fitting of cochlear implants (CI), increasing the benefit experienced by the CI users and leading to new perspectives in the development of smart implantable neurostimulators. 3D middle-ear reconstructions created after manual segmentation and related SM accessibility metrics were evaluated before the CI surgery for 16 candidates with assessed stapedius reflex. Retrofacial approach to access the SM was performed after facial recess exposure. In cases of poor exposition of SM, the access was performed anteriorly to the FN via drilling of the pyramidal eminence (PE). The total access rate of the SM via both the retrofacial and anterior approach of the FN was 100%. In 81.2% of cases (13/16), the retrofacial approach allowed to access the SM on previously categorized well exposed (8/8), partially exposed (4/5), and wholly concealed (1/3) SM with respect to FN. Following intraoperative evaluation in the remaining 18.8% (3/16), the SM was accessed anteriorly via drilling of the PE. Exposure of SM with respect to the FN and the sigmoid sinus’s prominence was a predictor for the suitable surgical approach. The retrofacial approach offers feasible and reproducible access to the SM belly, opening direct access to electromyographic sensing of the eSRT. Surgical planner tools can quantitatively assist pre-surgical assessment.

## Introduction

The reliable definition of the dynamic range for cochlear implant (CI) patients is a topic that is raising increasing interest in both clinical and medical technology environments. This interest is motivated by improving CI performance by optimizing the setting of all its tunable features. Among all the adjustable parameters, a critical one to ensure successful hearing rehabilitation is the maximum level of electrical charge delivered by the CI to elicit a maximum comfortable hearing sensation (MCL) in the user [1, 2]. The standard clinical routine for the MCL definition involves an active and compliant collaboration of the users providing their feedback during several fitting sessions [3, 4]. The main drawback of such behavioral-based fitting procedures is the intrinsic subjectivity of the CI users’ answers or their limited and variable ability to actively participate in the behavioral fitting process (e.g., small implanted children or cognitively impaired patients). This affects the reliability and reproducibility of the final outcome [4–9]. The stapedius reflex (SR) is an involuntary contraction of the stapedius muscle (SM) elicited in response to loud sound stimuli. In CI users, the SR is electrically evoked (eSR) via high levels of stimulation.

Interestingly, the eSR response has a high correlation with the MCL [1, 10–14]. Thus, the early detection of an eSR response—hereafter called eSR threshold (eSRT)—can objectively define the MCL, potentially replacing or complementing the behavioral fitting. The current clinical standard to record the stapedius reflex is non-invasive detection of the related admittance-induced changes in the outer ear canal. This procedure represents an indirect measure of the SM activity [15] and excludes cases of middle-ear malformations or conditions of compromised biomechanics involving ossicular chain, including the middle ear muscles (e.g., missing stapedius tendon) [16, 17]. In our previous study [18] we described a safe and reproducible surgical approach to directly access the SM tissue in a temporal bone (TB) study. This surgical approach is supported by virtual 3D reconstruction of the temporal bone and allows exposure of part of the SM belly by a posterior, retrofacial access. The exposed SM provides a route for directly measuring electromyographic muscle activity (EMG) and, therefore, reliable intraoperative measurement of the eSRT. In the current multicentric study, we demonstrate the feasibility, reproducibility, and safety of such a surgical approach in the intraoperative cochlear implant situation. In addition, we also show the potential usefulness of image processing-based surgical planning tools in pre-surgical assessment. The predictive power of the planning tool is analyzed in relation to the preferred surgical approach, comparing the posterior -retrofacial versus the anterior approach.

## Methods

### Patient recruitment

The ethics approval from both of the institutional review boards of the two centers (Jena and Tübingen, Germany) involved in our study was obtained. The main inclusion criteria were as follows: (1) age ≥ 18 years; (2) need of a cochlear implantation; (3) measurable acoustical stimulation of the SR from the contralateral side. Sixteen patients (7 males, 9 females, 49.8 ± 14.2 years old) were recruited among candidates applying for CI implantation following the German clinical guideline for CI implantation [19]. A summary of the patients’ characteristics is reported in Table 1.

**Table 1:** Demographic data of patients participating in our study (column 1 to 4). Summary of the pre-surgical evaluation (column 5) and surgical approach performed to access the stapedius muscle (column 6).

ID	Gender	Age	Surgery side	Pre-OP Evaluation	Approach performed
**1**	M	31	Right	E	R
**2**	F	78	Left	E	R
**3**	F	57	Right	C	A
**4**	F	58	Left	P	R
**5**	F	71	Left	E	R
**6**	F	57	Left	E	R
**7**	M	34	Left	C	R
**8**	M	41	Right	P	R
**9**	M	57	Left	C	A
**10**	M	67	Right	E	R
**11**	F	57	Right	E	R
**12**	F	40	Left	E	R
**13**	M	37	Right	P	R
**14**	F	38	Right	E	R
**15**	M	57	Left	P	A
**16**	F	33	Right	P	R

A: Anterior approach; C: Concealed stapedius muscle; E: Exposed stapedius muscle; P: Partially exposed stapedius muscle: R: Retrofacial approach

### Audiological screening

All the recruited patients underwent audiological screening. Both type A tympanogram and the presence of stapedius reflex were assessed (eTymp USB, Biomed Jena, in Jena and AT235, Interacoustics, Dortmund, in Tübingen). The pressure applied to the external auditory canal for the tympanometry ranged between -300 daPa and +200 daPa. Standard acoustic stimulation for stapedius reflex measurement was used (device probe tone frequency: 226 Hz, acoustic stimuli frequency: 500, 1000, 2000, 4000 Hz, acoustic stimuli intensity ranging from 80 to 100 dB, increasing step of 5 dB, stimulation duration 500 ms).

### Imaging acquisition and 3D segmentation

Imaging and 3D segmentation were performed for all 16 participants. Nine image datasets were collected at the Jena University Hospital the Department of Radiology within the routine clinical examination. System used was Artis Zeego Q system (Siemens Healthineers, Erlangen, Germany). Volumetric data were acquired with a single rotation of the C-arm mounted flat-panel detector cone-beam computed tomography (CT) system and reconstructed using Dyna-CT 3D dataset consisting of 400–550 slices (512 × 512 spacing; slice thickness 0.2 mm, slice separation 0.5 mm, voxel size 0.2 × 0.2 × 0.2 mm). The remaining seven datasets were collected at the Tübingen University Hospital. At the Tübingen site, scans were acquired using a third-generation single-source CT (SOMATOM^®^ Definition AS+, Siemens Healthineers, Erlangen, Germany) with a fully integrated circuit detector (Stellar^®^ detector, Siemens Healthineers, Erlangen, Germany). The imaging parameters were as follows: gantry rotation time, 1.0 second; tube current, 170 reference mAs using an automatic tube current modulation (CARE Dose4D^®^, Siemens Healthineers, Erlangen, Germany); tube voltage, 120 kV; yielding an average CT dose index (CTDIvol) of 20.1; and pitch, 0.85. The effective detector collimation was 16 × 0.3 mm using a z-axis UHR and flying focal spot technique, resulting in an effective 0.4-mm slice thickness. The scans were reconstructed using ADMIRE (Siemens Healthineers, Erlangen, Germany) at L3 strength level for the corresponding bone kernel.

Segmentation of all the datasets was manually performed using a standardized method, as described in [19]. The anatomical structures segmented were SM, intratemporal portion of the facial nerve (FN), chorda tympani (ChT), ossicular chain (OS), cochlea and vestibular organs (CV), sigmoid sinus (SS), and temporal bone (TB). 3D rendering was automatically generated for every patient based on the segmentation. The segmentation of one dataset took on average two hours.

### Surgery planning and accessibility evaluation

Expert otologic surgeons analyzed the 3D reconstructions during the pre-surgical planning session. The evaluation of the SM accessibility according to criteria described in [19] was performed (Table 1, column 5). In 5 cases, ID: #5, #7, #8, #9, and #16, to provide more information to the surgeons during the pre-surgical evaluation, the 3D reconstructions of each patient were fed into the recently developed surgical planning tool [20]. The surgical planning tool took on average 30 min to process one dataset and output the results. Output parameters such as the optimal head orientation, as well as distances between the hypothesized access spot and specifics landmarks such as FN, SS, VS were therefore considered to evaluate the surgical accessibility of the SM qualitatively. In the remaining cases, the surgical planning tools were used retrospectively, and such metrics as mentioned above were extracted post-operatively. For optimal head orientation, the patient’s head was rotated until the intraoperative situs matched to the image of the planning tool. See Table 2 for a summary of the results.

**Table 2:** Accessibility metrics extracted from the surgical planning tool developed in [20].

Patient	SM Exposed Area	Distance SM-FN	Distance SM-SS	Distance SM-VS	Depth of SM behind FN	Optimal Rotation	Optimal Head Tilt	DSC	Percentage Feasible Trajectories	Pre-OP Evaluation	Approach Performed
**1**	40.79	1.00	3.94	3.78	0.74	2	0	23.2	0.64	E	R
**2**	37.08	0.82	1.48	3.66	1.26	0	-26	4.4	0.06	E	R
**3**	29.84	0.55	12.64	2.37	3.02	6	16	2	0.13	C	A
**4**	25.23	0.89	5.07	5.42	1.08	-2	-8	19.2	0.38	P	R
**5**	49.79	1.05	3.32	3.52	1.97	0	-20	16	0.73	E	R
**6**	58.35	1.37	10.13	1.32	2.26	0	-24	26	0.52	E	R
**7**	0.00	N.a.	N.a.	N.a.	N.a.	N.a.	N.a.	0	0.00	C	R
**8**	15.63	0.70	5.60	1.98	1.57	0	-12	3.2	0.11	P	R
**9**	45.86	1.08	8.57	3.76	1.55	-2	0	12.4	0.49	C	A
**10**	57.44	1.19	3.10	2.98	1.33	0	0	13.2	0.77	E	R
**11**	79.02	0.92	9.08	3.44	1.65	2	0	8.8	0.59	E	R
**12**	59.44	1.15	5.47	3.31	1.01	2	0	16.4	0.76	E	R
**13**	98.29	0.97	1.19	3.33	0.26	0	0	6.8	0.56	P	R
**14**	73.19	1.19	4.60	2.09	0.86	0	0	24.4	0.81	E	R
**15**	36.58	1.08	6.94	4.82	2.36	0	24	9.6	0.37	P	A
**16**	49.65	0.85	2.55	2.03	0.27	-8	-14	8.8	0.29	P	R

Abbreviations: A: anterior approach, E: exposed SM, C: Concealed SM, DSC: Diameter Surgical Corridor, FN: Facial Nerve, N.a.: not available, P: partially exposed SM, R: retrofacial approach, SM: Stapedius muscle, SS: Sigmoid sinus, VS: Vestibular system. All the distances are expressed in millimeters. All the areas are expressed in mm^2^.

### Surgical procedure

Two experienced otologic surgeons (O.G.L and H.L) conducted the surgeries. For standard CI surgery, the procedure started with a standard mastoidectomy and posterior tympanotomy to reveal the head of the stapes and allow the identification of the stapedius tendon. Standard two-channel facial nerve monitoring was used. Before starting to drill the access to the SM, the presence of the stapedius reflex elicited by contralateral acoustic stimulation was visually verified by the surgeon (eTymp, BioMed, Jena, Germany; device probe tone frequency: 226 Hz, acoustic stimuli frequency: 500, 1000, 2000, 4000 Hz, acoustic stimuli intensity ranging from 80 to 110 dB, increasing step of 5 dB, 500 ms stimulation duration). The in-ear probe for measuring the stapedius reflex was inserted into the contralateral ear at the beginning of the surgery before the sterilization process.

**Fig 1:**
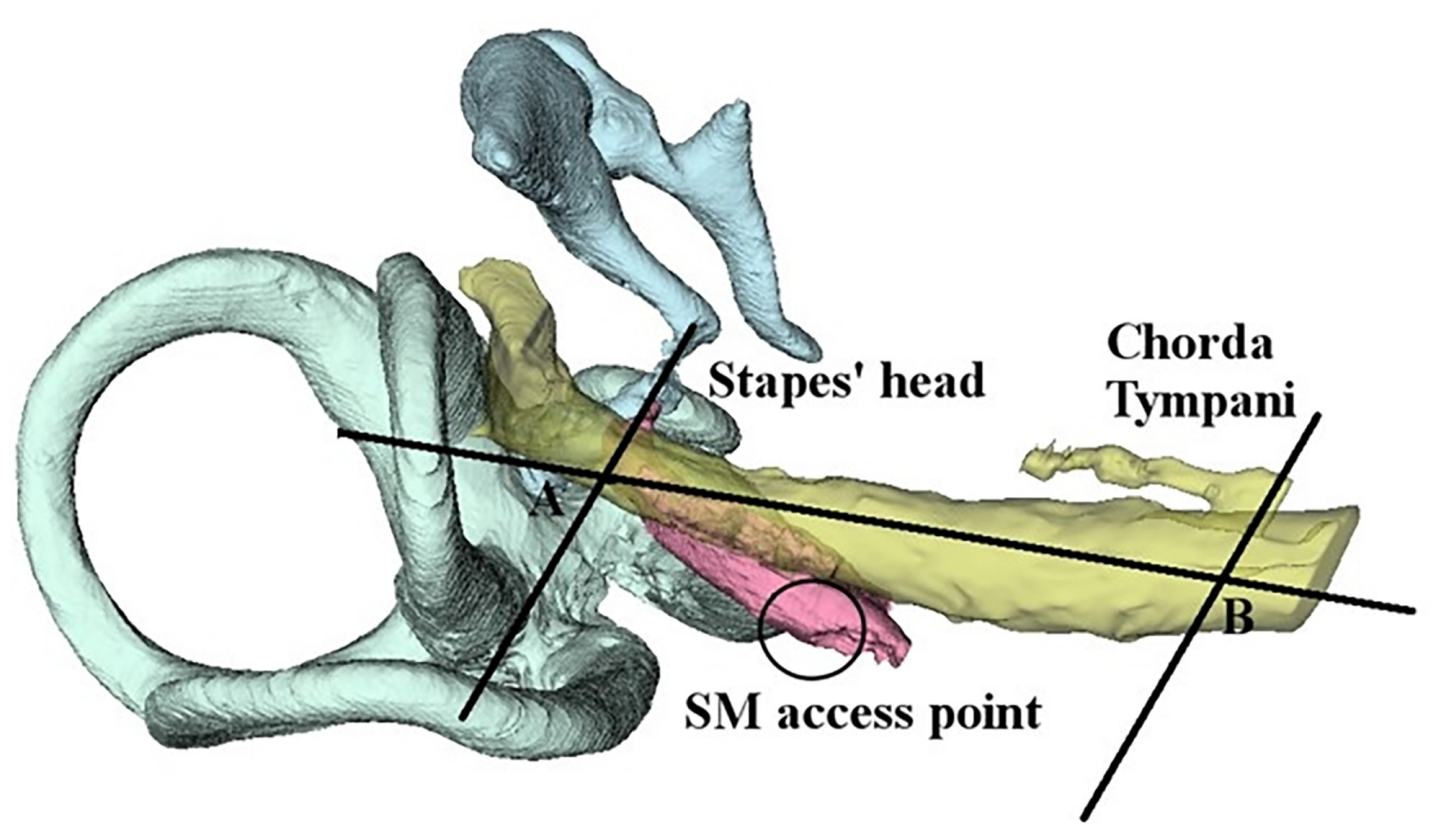
Schematic representation of the drilling spot identification to access the SM on the right side. A and B are the two extreme points of the segment running between the stapes’ head-tendon line (A) and the chorda tympani branching point (B), respectively. Adapted from [18].

The surgical access to the belly of the SM was drilled based on our previous results [18, 20, 21]. The access was performed posterior and medial to the mastoid portion of the FN, almost halfway between the stapes head level to the branching point of the chorda tympani (ChT) along the FN direction (Fig 1). The 3D reconstruction visually aligned with the actual position of the patient’s head was available for consultation during the whole duration of the surgery. In three cases (see Table 1), the muscle was accessed from the anterior to the FN by drilling the access on the PE. Such change related to the access strategy to SM was decided intraoperatively by the surgeon, once verified that the SM exposition with respect to the FN was insufficient for performing the retrofacial (posterior) approach. In addition to the previously described steps, neuromonitoring of the FN was constantly performed to avoid injuries of this structure. In the cases where the retrofacial approach was accomplished, to verify the access to the SM, the facial nerve (FN) monitoring device was used to stimulate the uncovered part of the SM, and careful visual observation of the stapes movement and SM movement through the microscope was carried out by the surgeon. Once the learning curve of the SM exposure was achieved, the additional duration added to the standard CI surgery was 20 to 30 min. The follow-up of the patients within the study ended on average 4–5 days after surgery with the discharge from the hospital.

### Exploratory data analysis

A secondary aim emerged during this work. This encompassed determining the potential of the image processing-based surgical planning tool in the decision process selecting the surgical approach. Nine accessibility metrics provided by the surgical planning tool (*SM Exposed Area; Distance SM-FN; Distance SM-SS Distance SM-VS; Depth of SM behind FN; Optimal Rotation; Optimal Head Tilt; Dimension Surgical Corridor (DSC);* and *Percentage of Feasible Trajectories*) were analyzed to investigate whether any significant difference existed between the two groups whose SM was accessed with different surgical approaches. Average, standard deviation and median of all the outcome metrics were computed among cases accessed with the same surgical approach (Table 3), and standard Wilcoxon’s sum rank tests were performed [21].

**Table 3:** Averaged accessibility metrics among cases accessed with the same surgical approach and statistical comparison between the two surgical approaches. Metrics are extracted from the surgical planning tool developed in [20].

Parameter	Retrofacial Approach (13 patients)			Anterior Approach (3 cases)			Wilcoxon Rank Sum
Accessibility metrics	Mean	Median	Std.Dev.	Mean	Median	Std.Dev.	p-value
SM Exposed Area	49.53	49.79	25.53	37.43	36.58	6.57	0.2964
Distance SM_FN	1.01	0.99	0.18	0.90	1.08	0.25	0.8396
Distance SM_SS	4.63	4.27	2.62	9.38	8.57	2.39	**0.0484**
Distance SM_VS	3.07	3.32	1.05	3.65	3.76	1.00	0.3648
Depth of SM behind FN	1.19	1.17	0.59	2.31	2.36	0.60	**0.0484**
Optimal Rotation	-0.33	0.00	2.56	1.33	0.00	3.40	0.8703
Optimal Head Tilt	-8.67	-4.00	9.81	13.33	16.00	9.98	**0.0308**
DSC	13.11	13.20	8.20	8.00	9.60	4.39	0.4214
Percentage Feasible Trajectories	0.48	0.56	0.27	0.33	0.37	0.15	0.3643

Abbreviations: A: anterior approach, E: exposed SM, C: Concealed SM, DSC: Diameter Surgical Corridor, FN: Facial Nerve, N.a.: not available, P: partially exposed SM, R: retrofacial approach, SM: Stapedius Muscle, SS: Sigmoid Sinus, Std.Dev. = Standard deviation, VS: Vestibular System. All the distances are expressed in millimeters. All the areas are expressed in mm^2^. P-values ≤0.05 are reported in bold.

## Results

**Fig 2:**
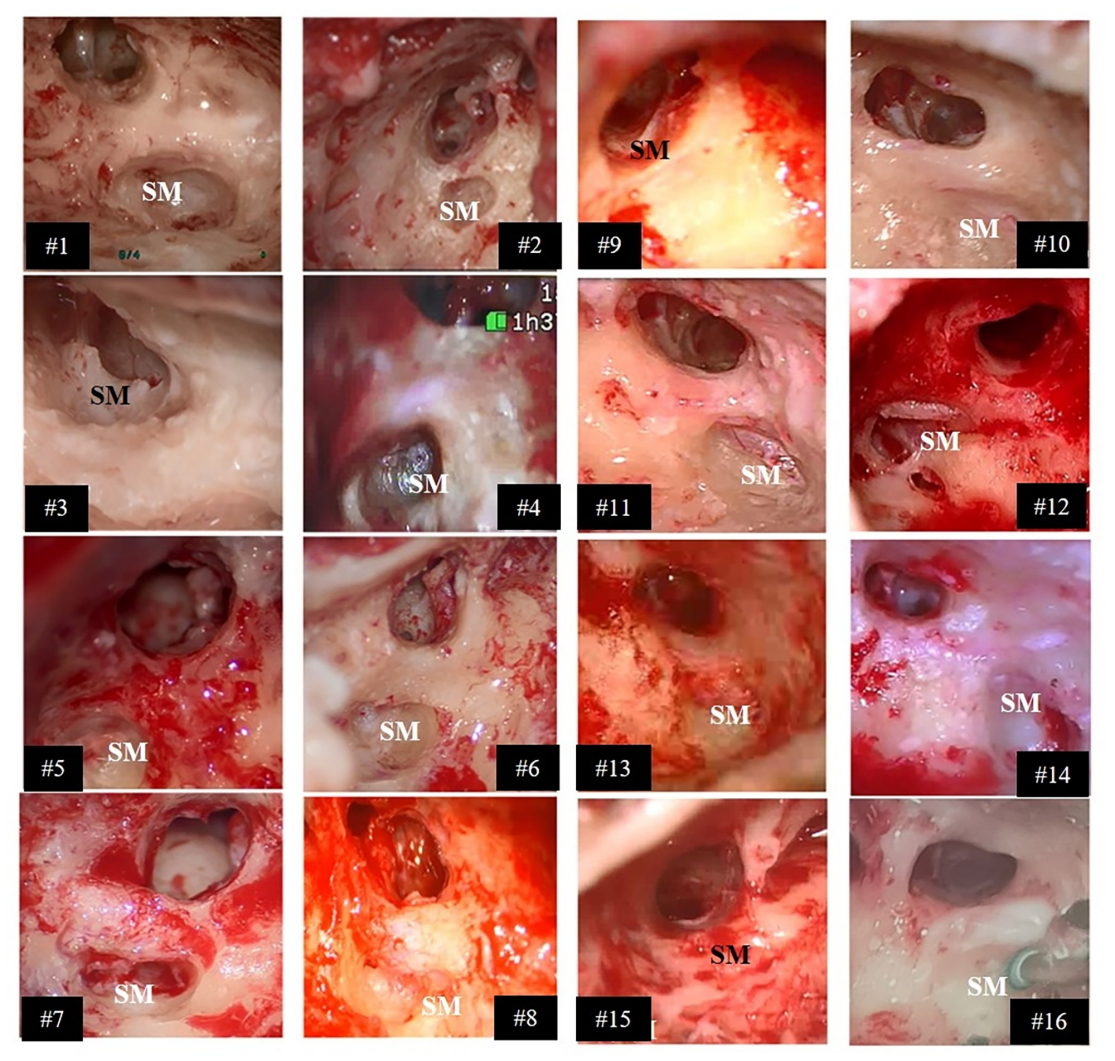
Access to the SM drilled on the sixteen patients. Screenshots taken from intraoperative microscope recordings. Patients #3, #9, and #15: anterior approach; all other patients: retrofacial approach. In #16, an electrostimulation probe is placed on the stapedius muscles.

The belly of the SM was successfully accessed via the retrofacial (posterior) approach on 13 (out of 16) patients, relating to a total success rate of 81.2% (Table 1). In the remaining 3 cases the SM muscle was accessed on via an anterior approach by drilling of the PE. In the retrofacial approach, the surgical spot was drilled posterior and medial to the mastoid portion of the FN at almost half-way between the identified level of the stapes head and the branching out of the ChT. A screenshot reporting the surgical access drilled for every participant is shown in Fig 2.

**Fig 3:**
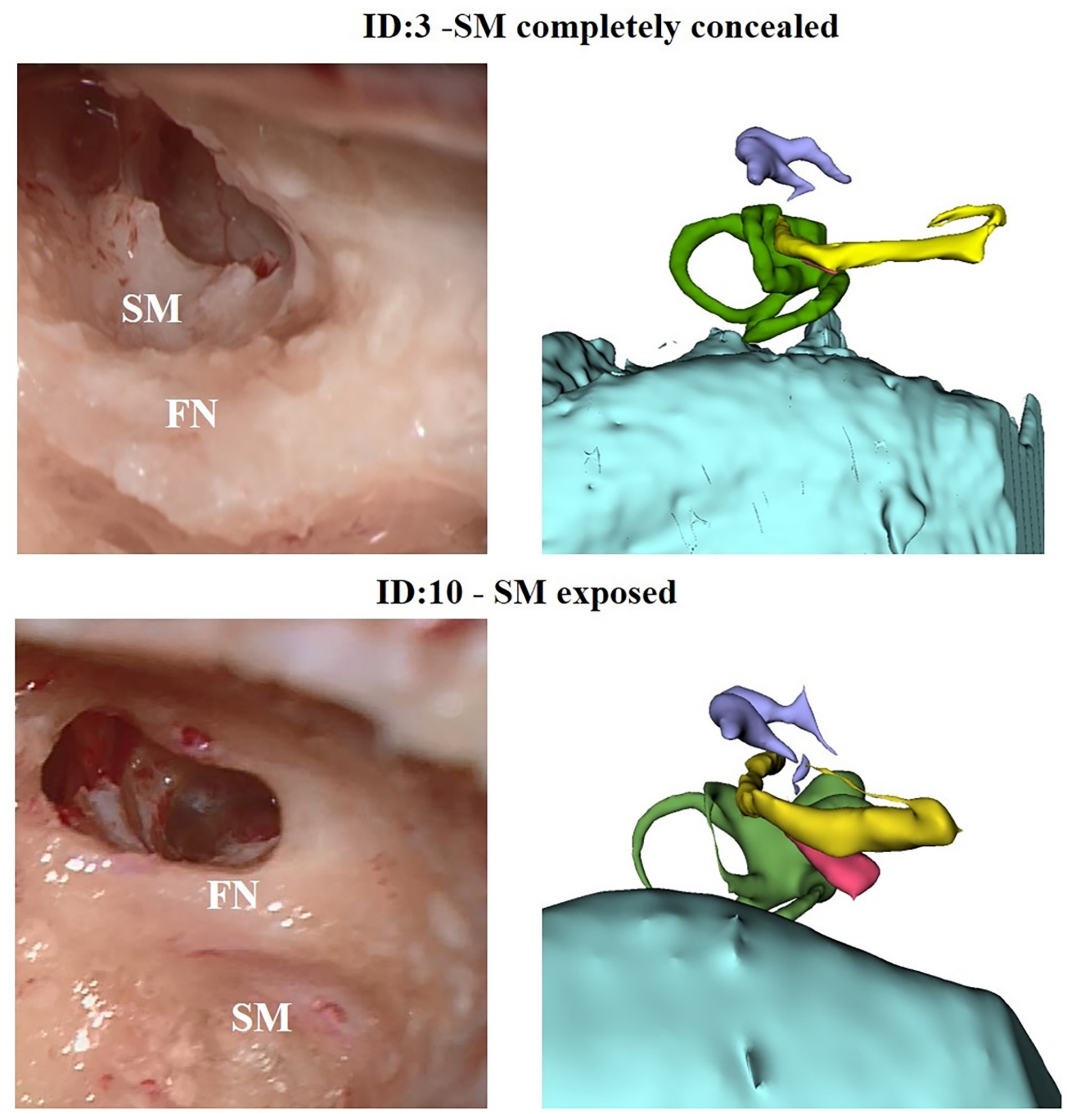
Comparison between two different SM configurations leading to two different surgical accesses, right ear. Top: case #3 categorized as *SM completely concealed* (according to [19]). Bottom: case #10 categorized as *SM exposed* (according to [19]). Surgical microscopic view of the access performed from anterior the FN (left) and 3-D reconstruction (right). SM: stapedius muscle, FN: facial nerve.

**Fig 4:**
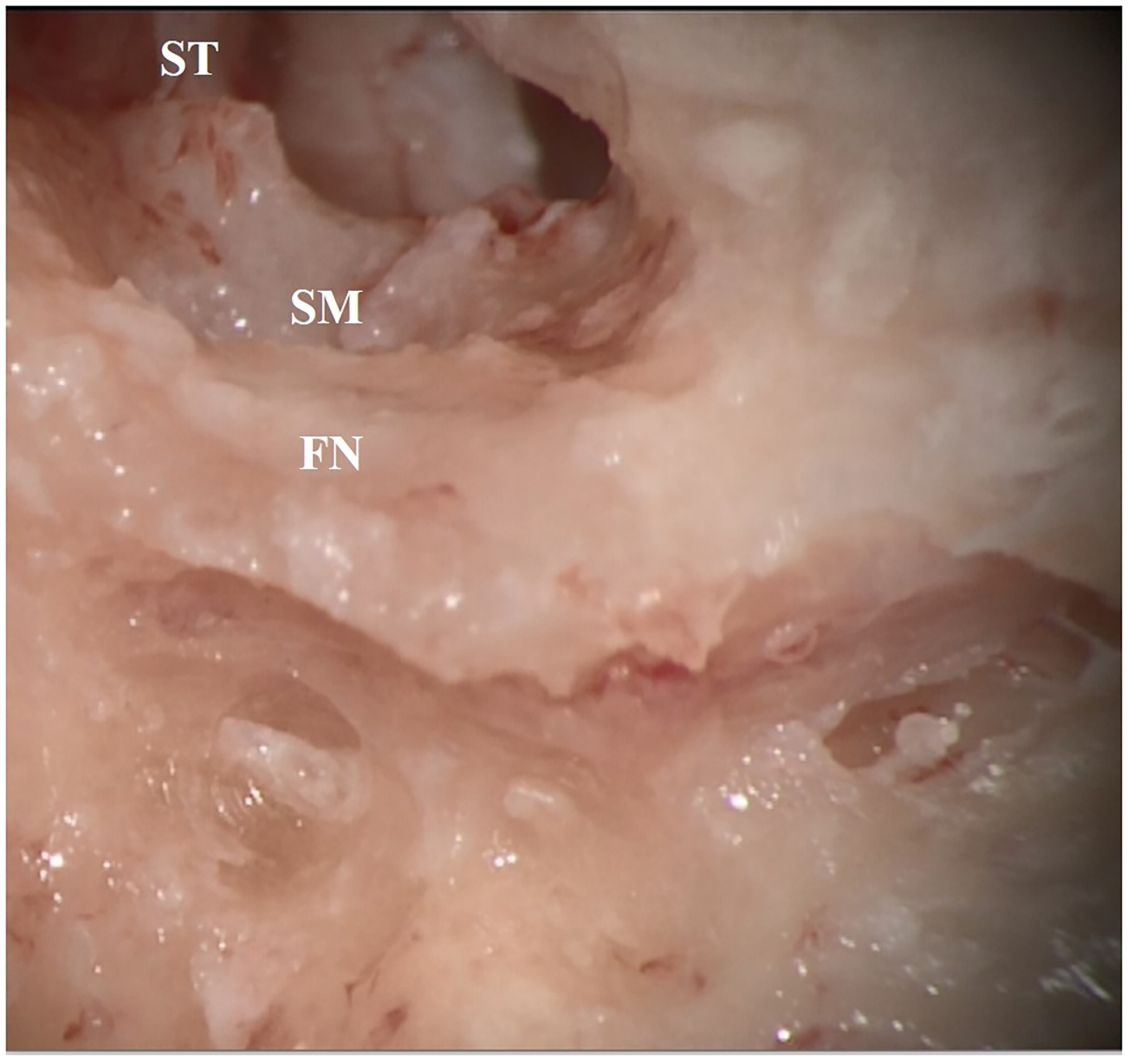
Higher magnification of the upper case of Fig 3, right ear. Surgical view form the microscope preceding the decision of the surgeon of switching from the retrofacial approach to the anterior approach with drilling of the pyramidal eminence (PE) to access the SM. After clear and deep exposition of the FN course, the SM appeared out of reach through the retrofacial approach. Therefore, the surgical approach was changed towards an anterior approach. Screenshot taken from intraoperative microscope recordings. FN: facial nerve, SM: stapedius muscle, ST: stapedius tendon.

**Fig 5:**
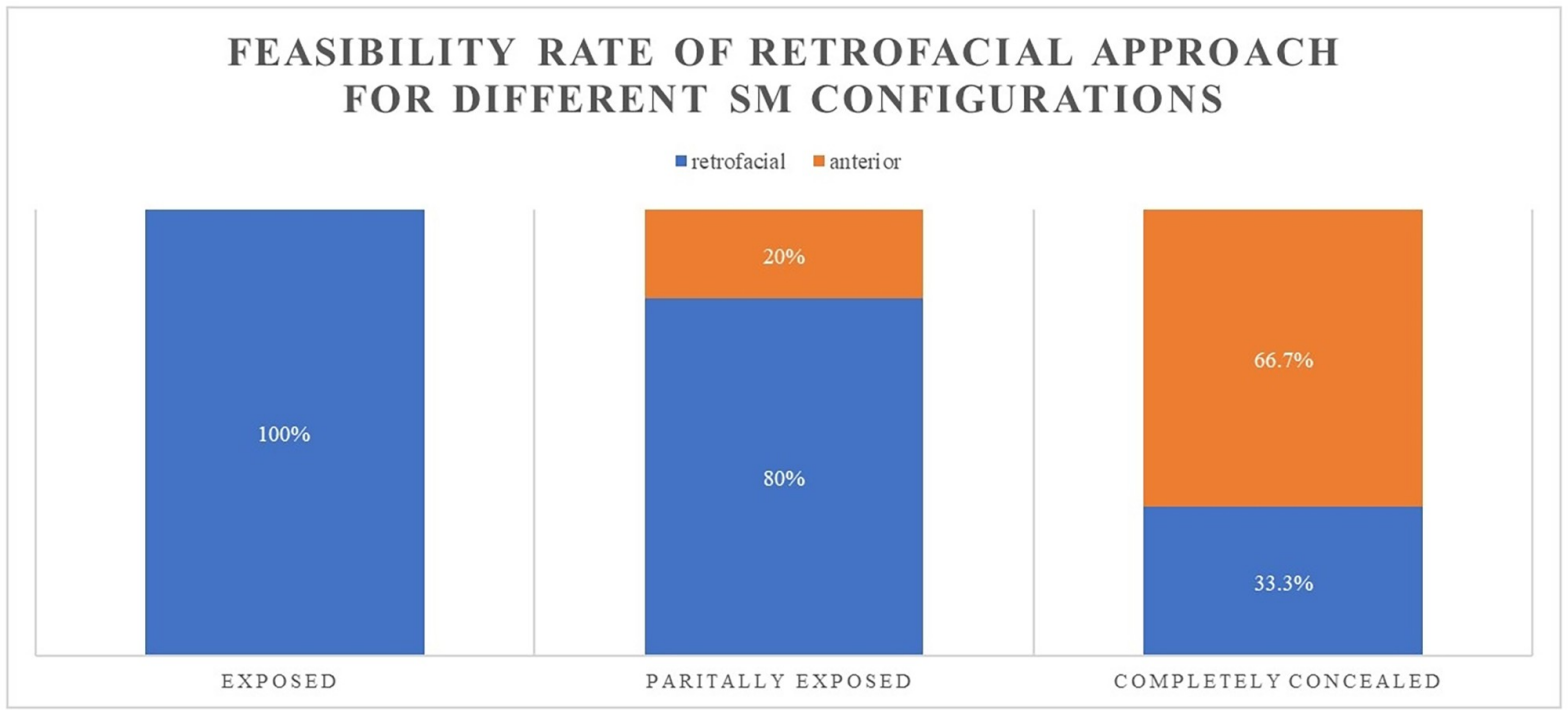
Bar chart summarizing the feasibility/success rate of the retrofacial approach in different configurations of the stapedius muscle respect to the facial nerve (according to [19]).

In 100% of the cases (8 out of 8) for which the SM was pre-surgically categorized as *exposed* with respect to FN, the retrofacial approach was successful and the access to the muscle was gained smoothly. In 80% of the cases (4 out of 5) for which the SM was pre surgically categorized as *partially exposed*, the retrofacial approach successfully reached the SM. In 33.3% of cases (1 out of 3) for which the SM was pre surgically categorized as *completely concealed*, the retrofacial approach still reached the SM. A magnified example of two different SM configurations that led to the two different surgical approaches are shown in Fig 3—left. Their relative 3D reconstructions showing the difference of the SM exposition are reported in Fig 3—right. In Fig 4, one example of the final surgical view before the intraoperative decision of the surgeon of switching from retrofacial access of the SM to anterior drilling of the PE due to poor exposition of the SM respect to FN is reported. In Fig 5, a summary of the feasibility rate in the three different configurations of the SM with respect to FN is reported. In Table 3, the results related to our exploratory data analysis on the metrics produced via the surgical planning tool are reported. Among all the comparisons, for three metrics (Distance SM_SS, p = 0.0484; Depth of SM behind FN, p = 0.0484; *Optimal Head Tilt*, p = 0.0308), a significant difference (p-value ≤0.05) between the two groups (Retrofacial approach vs. anterior approach) was observed.

At the end of the surgery, the SR, stimulated on the contralateral side and measured on implantation side, disappeared but recovered during follow-up 6 weeks later. One patient had a transient postoperative facial palsy that recovered with one month. No other complications were seen.

## Discussion

In the current work we demonstrated the feasibility, reproducibility, and safety of accessing the SM via the retrofacial approach in CI surgery patients. No major complications occurred. Especially, the rate of transient postoperative facial palsy was in the expected range and no permanent facial palsy occurred. The retrofacial approach is a well-known technique performed in otologic surgery for cholesteatoma removal from the sinus tympani [22, 23], for CI implantation on malformed anatomies [24, 25] and in cases of poor visibility of round windows [26] and for middle ear implant surgeries [27, 28]. Still, to our knowledge, there is no literature discussing its use for accessing the SM.

The high feasibility rate of the retrofacial approach to access the SM in the current data set is comparable and superior to the one achieved on human TBs [18]. This result validated the crucial importance of practical training on cadaveric samples [29]. Furthermore, the results provided additional and evidence obtained during routine CI surgery on the reproducibility of the approach despite the reported anatomical variability of the human SM [30]. As expected, the location of the drilling access point to the SM was found medial and posterior with respect to the mastoid tract of the FN at halfway between the SM tendon level and the potential branching point of the ChT. This outcome is in line with the results we have obtained from our previous anatomical TBs study [18]. Moreover, the anatomical landmarks considered intraoperatively such as the mastoid tract of the FN, the stapedial tendon, the height/prominence of the sigmoid sinus confirm our previously described anatomical benchmarks, supporting the formulation of a reproducible surgical approach.

From the surgical point of view, the role of the FN monitoring device appears quite crucial. In fact, besides providing continuous feedback to the otologic surgeons on the anatomic course of the FN reducing potential nerve damage [31], it offered them a validation tool to verify the successful access to the SM belly visually. The availability of the 3D reconstruction of the individual patient anatomy during the surgery session and the possibility to dynamically visualize different orientations of the SM is important. The presented results confirm that this is a powerful support for improving the outcome of the access [32].

The accessibility to the 3D anatomical information of the individual patient was also relevant during pre-surgical planning. This relevance is quantitatively supported by the 100% correspondence between the cases pre-surgically categorized as “exposed” and the related successful access of the SM via the retrofacial approach. Conversely, in 66.6% of the patients pre-surgically classified as “completely concealed” the intraoperative anatomical situation led the surgeon to reconsider the more convenient the anterior access to the SM via drilling of the PE. In accordance with our previous results [19], we confirm that the evaluation of 3D rendering is a helpful and advantageous tool for the preoperative planning of eSRT measurements from the SM via the retrofacial approach.

The alternative anterior access to the SM—via drilling of the PE and through the stapedial tendon to record the electromyographic activity of the SM have been previously documented [33–35]. This anterior type surgical approach might decrease the success rate regarding access to the muscle fibers. On the other hand, the anterior approach limits the exposition of critical structures such as FN to potential damage. In fact, according to histological results in human fetuses [36, 37], the superior compartment part of the stapedial cavity accessed by the anterior approach contains primarily tendinous tissue. In contrast, the inferior compartment of the stapedial cavity accessed by the posterior, retrofacial approach contains the stapedial muscle fibers that also originate in this compartment. Therefore, the retrofacial approach offers a more direct access to the source of the EMG signal. Interesting and promising results have been generated in our exploratory data analysis supporting the relevance of pre-surgical planning tools in the surgical decision process. Although the surgical planning tool used in the current study is still in its validation phase, it was possible to extract—among the provided metrics—three potential predictors that correlate with the use of the most suitable surgical approach. The degree of the prominence of the sigmoid sinus in relation to the surgical view over the SM target (expressed in our case by the metric *Distance SM_SS*) appears to be a significant discriminating factor for the surgical decision. A shorter planar distance between the two structures limits the surgical corridor available to the surgeon. The limited surgical corridor increases the risk of FN injury in the retrofacial approach and therefore is less feasible. This conclusion is similar to CI surgery [26, 38], where the degree of prominence of the sigmoid sinus appeared to be a crucial factor for the feasibility of the retrofacial approach. The level of anterior displacement of the SM in relation to the course of the FN (expressed by the metrics *Depth SM behind FN*) was shown to be a further valid predictor for the surgical approach. The more the position of SM is found anterior and more parallel to the FN, the higher is its degree of concealment. In this situation, the surgeon needs to drill more in the proximity of the FN, which increases the complexity and risk of the procedure. Since the SM is not a specific target of retrofacial approaches, its anatomical characteristics have not been previously discussed in the literature in this context. The third relevant discriminating metric is the degree of tilting (expressed by *Optimal Head Tilt*) of the patient’s head from a standard position during execution of posterior tympanotomy. The more the surgeon needs to tilt the patient’s head to reach the target, the more the sigmoid sinus is shifted anteriorly, thus hindering the drilling trajectory for the retrofacial approach. This result has been discussed regarding of sigmoid sinus prominence (see above).

The current standard for intraoperative eSRT measurements is the visual identification of the SR by the surgeon. Unfortunately, this method is highly subjective and hampered by ventilation-associated situs excursions, blinking, or other distractions [39]. Recently, Weiss et al. presented a tracking software to quantify stapes head movements automatically [39]. So far, the system was tested postoperatively against visual registration on the video material. We want to go one step further by recording of a needle EMG of the SM during the eSRT measurement as the absolute proof of an activation of the SR. Such recordings are in the next clinical trial. Furthermore, the ultimate goal is to permanently implant this recording electrode in the SM like implanting the CI in the cochlea. This would allow an objective and long-term eSRT fitting of the CI.

The limitations of our study are mainly related to the exploratory format of the analysis; the sample size limits the statistical analysis and different datasets need to be included to increase the statistical power of our results. Furthermore, only adult patients were analyzed. Further trials have to verify that the approach is also feasible in children. In children, also the pros and cons of an additional 3D reconstruction based on CT data has to be weighed against the radiation exposure. In the present study, we used a low-dose DYNA-CT protocol. Moreover, a longer follow-up period is needed to evaluate if the exposure of the SM induces fibrosis that could impair the SR long term preservation.

## Conclusions

In the presented study, the feasibility of directly accessing the SM via the retrofacial approach was demonstrated intraoperatively during routine CI surgery. This achievement opens up new options to intraoperatively measure the eSRT and to incorporate this knowledge into future smart CI systems. The execution of the surgery—based on the same anatomical landmarks used in our previous anatomical TBs study—confirms the reproducibility and safety of the approach. The pre-surgical evaluation and categorization of 3D reconstruction of middle ear anatomy proved a valuable tool for otologic surgeons empowering the planning of the most suitable drilling strategy. The outcome metrics showed that the surgical planner tool helps to predict the best possible surgical approach and access to the SM. This outcome advocates the further validation of the surgical planning tool. In general, it substantiates the idea of developing tools to assist surgeons in the pre-surgical decision process.

## Supporting information

S1 File (DOCX)
