# Supplementary material for: Accessing the stapedius muscle via novel surgical retrofacial approach during cochlear implantation surgery: Intraoperative results on feasibility and safety
Source: PLoS One. 2022 Aug 11;17(8):e0272943. doi: 10.1371/journal.pone.0272943 (PMC9371293; doi:10.1371/journal.pone.0272943)
Supplement: S1 File — (DOCX) [file pone.0272943.s001.docx]

**Minimal anonymized data set of the 16 patients**

**Accessing the stapedius muscle via novel surgical retrofacial approach during cochlear implantation surgery: intraoperative results on feasibility and safety**

Orlando Guntinas-Lichius^1*^, Dirk Arnold^1^, Gerd Fabian Volk^1^, Daniela Korth^1^, Rene Aschenbach^2^, Johann-Martin Hempel^3^, Fritz Schneider^4^, Thore Schade-Mann^4^, Philipp Gamerdinger^4^, Anke Tropitzsch^4^, Hubert Löwenheim^4^

^1^Department of Otorhinolaryngology, Jena University Hospital, Jena, Germany

^2^Department of Radiology, Jena University Hospital, Am Klinikum 1, 07747 Jena, Germany

^3^Department of Neuroradiology, University of Tübingen Medical Center, Tübingen, Germany

^4^Department of Otolaryngology-Head & Neck Surgery, Hearing Research Center, University of Tübingen Medical Center, Tübingen, Germany

**Demographic data of patients, intra- and postoperative data**

| **ID** | **Gender** | **Age** | **Surgery side** | **Pre-OP Evaluation** | **Approach performed** | **Pre-OP**  **SR** | **Intra-Op**  **Complication** | **Additional time for SM exposure** | **Post-OP**  **Complication until discharge** | **Comments** | **Particularities** |
| --- | --- | --- | --- | --- | --- | --- | --- | --- | --- | --- | --- |
| **1** | M | 31 | Right | E | R | Yes | No | <20 min | No |  |  |
| **2** | F | 78 | Left | E | R | Yes, weak | No | <20 min | No |  |  |
| **3** | F | 57 | Right | C | A | Yes | No | <30 min | Transient facial palsy | Resolved within 4 weeks |  |
| **4** | F | 58 | Left | P | R | Yes | No | <30 min | No |  |  |
| **5** | F | 71 | Left | E | R | Yes | No | <40 min | No |  | Cochlear calification |
| **6** | F | 57 | Left | E | R | Yes | No | unknown | No |  |  |
| **7** | M | 34 | Left | C | R | Yes | No | <20 min | No |  | SR disappeared at the end of surgery |
| **8** | M | 41 | Right | P | R | Yes | No | <20 min | No |  |  |
| **9** | M | 57 | Left | C | A | Yes | No | <20 min | No |  |  |
| **10** | M | 67 | Right | E | R | Yes | No | <15 min | No |  |  |
| **11** | F | 57 | Right | E | R | Yes | No | <20 min | No |  |  |
| **12** | F | 40 | Left | E | R | Yes, weak | No | <20 min | No |  |  |
| **13** | M | 37 | Right | P | R | Yes | No | <30 min | No |  |  |
| **14** | F | 38 | Right | E | R | Yes | No | <20 min | No |  |  |
| **15** | M | 57 | Left | P | A | Yes | No | <20 min | No |  |  |
| **16** | F | 33 | Right | P | R | Yes | No | <20 min | No |  |  |
| **16** | F | 33 | Right | P | R | Yes | No | <20 min | No |  |  |

A: Anterior approach; C: Concealed stapedius muscle; E: Exposed stapedius muscle; P: Partially exposed stapedius muscle: R: Retrofacial approach

**Accessibility metrics extracted from the surgical planning tool**

| **Patient** | **SM**  **Exposed Area** | **Distance**  **SM-FN** | **Distance**  **SM-SS** | **Distance**  **SM-VS** | **Depth of SM behind FN** | **Optimal**  **Rotation** | **Optimal**  **Head Tilt** | **DSC** | **Percentage**  **Feasible Trajectories** | **Pre-OP**  **Evaluation** | **Approach**  **Performed** |
| --- | --- | --- | --- | --- | --- | --- | --- | --- | --- | --- | --- |
| **1** | 40.79 | 1.00 | 3.94 | 3.78 | 0.74 | 2 | 0 | 23.2 | 0.64 | E | R |
| **2** | 37.08 | 0.82 | 1.48 | 3.66 | 1.26 | 0 | -26 | 4.4 | 0.06 | E | R |
| **3** | 29.84 | 0.55 | 12.64 | 2.37 | 3.02 | 6 | 16 | 2 | 0.13 | C | A |
| **4** | 25.23 | 0.89 | 5.07 | 5.42 | 1.08 | -2 | -8 | 19.2 | 0.38 | P | R |
| **5** | 49.79 | 1.05 | 3.32 | 3.52 | 1.97 | 0 | -20 | 16 | 0.73 | E | R |
| **6** | 58.35 | 1.37 | 10.13 | 1.32 | 2.26 | 0 | -24 | 26 | 0.52 | E | R |
| **7** | 0.00 | N.a. | N.a. | N.a. | N.a. | N.a. | N.a. | 0 | 0.00 | C | R |
| **8** | 15.63 | 0.70 | 5.60 | 1.98 | 1.57 | 0 | -12 | 3.2 | 0.11 | P | R |
| **9** | 45.86 | 1.08 | 8.57 | 3.76 | 1.55 | -2 | 0 | 12.4 | 0.49 | C | A |
| **10** | 57.44 | 1.19 | 3.10 | 2.98 | 1.33 | 0 | 0 | 13.2 | 0.77 | E | R |
| **11** | 79.02 | 0.92 | 9.08 | 3.44 | 1.65 | 2 | 0 | 8.8 | 0.59 | E | R |
| **12** | 59.44 | 1.15 | 5.47 | 3.31 | 1.01 | 2 | 0 | 16.4 | 0.76 | E | R |
| **13** | 98.29 | 0.97 | 1.19 | 3.33 | 0.26 | 0 | 0 | 6.8 | 0.56 | P | R |
| **14** | 73.19 | 1.19 | 4.60 | 2.09 | 0.86 | 0 | 0 | 24.4 | 0.81 | E | R |
| **15** | 36.58 | 1.08 | 6.94 | 4.82 | 2.36 | 0 | 24 | 9.6 | 0.37 | P | A |
| **16** | 49.65 | 0.85 | 2.55 | 2.03 | 0.27 | -8 | -14 | 8.8 | 0.29 | P | R |

A: anterior approach, E: exposed SM, C: Concealed SM, DSC: Diameter Surgical Corridor, FN: Facial Nerve, N.a.: not available, P: partially exposed SM, R: retrofacial approach, SM: Stapedius muscle, SS: Sigmoid sinus, VS: Vestibular system. All the distances are expressed in millimeters. All the areas are expressed in mm^2^.
