# Supplementary material for: Accessing the stapedius muscle via novel surgical retrofacial approach during cochlear implantation surgery: Intraoperative results on feasibility and safety
Source: PLoS One. 2022 Aug 11;17(8):e0272943. doi: 10.1371/journal.pone.0272943 (PMC9371293; doi:10.1371/journal.pone.0272943)
Supplement: Peer review history

# Peer review: Decision Letter 0

**DOI:** 10.1371/journal.pone.0272943.r001

**Authors:** Adrien A. Eshraghi

23 Jun 2022

PONE-D-22-13880Accessing the stapedius muscle via novel surgical retrofacial approach during cochlear implantation surgery: intraoperative results on feasibility and safetyPLOS ONE

Dear Dr. Guntinas-Lichius,

Thank you for submitting your manuscript to PLOS ONE. After careful consideration, we feel that it has merit but does not fully meet PLOS ONE’s publication criteria as it currently stands. Therefore, we invite you to submit a revised version of the manuscript that addresses the points raised during the review process.

Please submit your revised manuscript by Aug 07 2022 11:59PM. If you will need more time than this to complete your revisions, please reply to this message or contact the journal office at plosone@plos.org. When you're ready to submit your revision, log on to https://www.editorialmanager.com/pone/ and select the 'Submissions Needing Revision' folder to locate your manuscript file.

Please include the following items when submitting your revised manuscript:

- A rebuttal letter that responds to each point raised by the academic editor and reviewer(s). You should upload this letter as a separate file labeled 'Response to Reviewers'.
- A marked-up copy of your manuscript that highlights changes made to the original version. You should upload this as a separate file labeled 'Revised Manuscript with Track Changes'.
- An unmarked version of your revised paper without tracked changes. You should upload this as a separate file labeled 'Manuscript'.

If you would like to make changes to your financial disclosure, please include your updated statement in your cover letter. Guidelines for resubmitting your figure files are available below the reviewer comments at the end of this letter.

If applicable, we recommend that you deposit your laboratory protocols in protocols.io to enhance the reproducibility of your results. Protocols.io assigns your protocol its own identifier (DOI) so that it can be cited independently in the future. For instructions see: https://journals.plos.org/plosone/s/submission-guidelines#loc-laboratory-protocols. Additionally, PLOS ONE offers an option for publishing peer-reviewed Lab Protocol articles, which describe protocols hosted on protocols.io. Read more information on sharing protocols at https://plos.org/protocols?utm_medium=editorial-email&utm_source=authorletters&utm_campaign=protocols.

We look forward to receiving your revised manuscript.

Kind regards,

Adrien A. Eshraghi, M.D.

Academic Editor

PLOS ONE

Journal Requirements:

When submitting your revision, we need you to address these additional requirements.

1. Please ensure that your manuscript meets PLOS ONE's style requirements, including those for file naming. The PLOS ONE style templates can be found at 

https://journals.plos.org/plosone/s/file?id=wjVg/PLOSOne_formatting_sample_main_body.pdf and 

https://journals.plos.org/plosone/s/file?id=ba62/PLOSOne_formatting_sample_title_authors_affiliations.pdf

2. Please provide additional details regarding participant consent. In the ethics statement in the Methods and online submission information, please ensure that you have specified what type you obtained (for instance, written or verbal, and if verbal, how it was documented and witnessed). If your study included minors, state whether you obtained consent from parents or guardians. If the need for consent was waived by the ethics committee, please include this information.

3. Thank you for stating the following financial disclosure: 

"The study was sponsored by MED-EL Elektromedizinische Geräte GmbH, Innsbruck, Austria."

Please state what role the funders took in the study.  If the funders had no role, please state: "The funders had no role in study design, data collection and analysis, decision to publish, or preparation of the manuscript." 

If this statement is not correct you must amend it as needed. 

Please include this amended Role of Funder statement in your cover letter; we will change the online submission form on your behalf.

4. Thank you for stating the following in the Acknowledgments Section of your manuscript: 

"The study was sponsored by MED-EL Elektromedizinische Geräte GmbH, Innsbruck, Austria."

We note that you have provided funding information that is not currently declared in your Funding Statement. However, funding information should not appear in the Acknowledgments section or other areas of your manuscript. We will only publish funding information present in the Funding Statement section of the online submission form. 

Please remove any funding-related text from the manuscript and let us know how you would like to update your Funding Statement. Currently, your Funding Statement reads as follows: 

"The study was sponsored by MED-EL Elektromedizinische Geräte GmbH, Innsbruck, Austria."

Please include your amended statements within your cover letter; we will change the online submission form on your behalf.

5. We note that you have indicated that data from this study are available upon request. PLOS only allows data to be available upon request if there are legal or ethical restrictions on sharing data publicly. For more information on unacceptable data access restrictions, please see http://journals.plos.org/plosone/s/data-availability#loc-unacceptable-data-access-restrictions. 

In your revised cover letter, please address the following prompts:

a) If there are ethical or legal restrictions on sharing a de-identified data set, please explain them in detail (e.g., data contain potentially sensitive information, data are owned by a third-party organization, etc.) and who has imposed them (e.g., an ethics committee). Please also provide contact information for a data access committee, ethics committee, or other institutional body to which data requests may be sent.

b) If there are no restrictions, please upload the minimal anonymized data set necessary to replicate your study findings as either Supporting Information files or to a stable, public repository and provide us with the relevant URLs, DOIs, or accession numbers. For a list of acceptable repositories, please see http://journals.plos.org/plosone/s/data-availability#loc-recommended-repositories.

We will update your Data Availability statement on your behalf to reflect the information you provide.

Additional Editor Comments:

Reviewers all presented with different opinions from rejection to minor revision. This paper is interesting ans presents an innovating and interesting surgical approach that could be beneficial for cochlear implant fittings. We suggest to accept with Major Revisions after authors properly adress the reviewers comments.

[Note: HTML markup is below. Please do not edit.]

Reviewers' comments:

Reviewer's Responses to Questions

**Comments to the Author**

1. Is the manuscript technically sound, and do the data support the conclusions?

The manuscript must describe a technically sound piece of scientific research with data that supports the conclusions. Experiments must have been conducted rigorously, with appropriate controls, replication, and sample sizes. The conclusions must be drawn appropriately based on the data presented. 

Reviewer #1: Yes

Reviewer #2: Yes

Reviewer #3: Yes

**********

2. Has the statistical analysis been performed appropriately and rigorously? 

Reviewer #1: Yes

Reviewer #2: N/A

Reviewer #3: No

**********

3. Have the authors made all data underlying the findings in their manuscript fully available?

The PLOS Data policy requires authors to make all data underlying the findings described in their manuscript fully available without restriction, with rare exception (please refer to the Data Availability Statement in the manuscript PDF file). The data should be provided as part of the manuscript or its supporting information, or deposited to a public repository. For example, in addition to summary statistics, the data points behind means, medians and variance measures should be available. If there are restrictions on publicly sharing data—e.g. participant privacy or use of data from a third party—those must be specified.

Reviewer #1: Yes

Reviewer #2: Yes

Reviewer #3: No

**********

4. Is the manuscript presented in an intelligible fashion and written in standard English?

PLOS ONE does not copyedit accepted manuscripts, so the language in submitted articles must be clear, correct, and unambiguous. Any typographical or grammatical errors should be corrected at revision, so please note any specific errors here.

Reviewer #1: Yes

Reviewer #2: Yes

Reviewer #3: Yes

**********

5. Review Comments to the Author

Please use the space provided to explain your answers to the questions above. You may also include additional comments for the author, including concerns about dual publication, research ethics, or publication ethics. (Please upload your review as an attachment if it exceeds 20,000 characters)

Reviewer #1: The authors investigated and compared the feasibility, reproducibility, and safety of two surgical approaches in cochlear implant surgery. The main aim of the study is to evaluate the stapedius muscle (SM) more objectively, via direct measurement from the muscle itself. With this hypothesis pre and retrofacial routes have been studied. Additionally, an image processing-based surgical planning tool in pre-surgical assessment was also investigated.

Virtual 3D reconstruction of the temporal bone has been used lately especially in the robotic cochlear implantation and also for resident training programmes.

The article, in general, is well written with very few grammatical errors.

In the methodology, the authors should specify the inclusion and exclusion criteria of the participants.

The main point of the discussion should focus on the benefit of SRT measurements directly form SM. The authors did not clarify this issue. Are there any investigations comparing the outcomes of traditional SRT mesurements with the direct SM SRT measurements on the CI fittings and tuning?

Although retrofacial approach can be used in selected CI cases, the rationale on choosing this approach should be more clarified. Maybe a preliminary evaluation on the benefits of SRT measurements directly from SM will make this study more understandle and clear.

Reviewer #2: This study report the safety of a new indication of the retrofacial approach to expose the stapedius muscle body. The goal of the study is to allow the stapedius reflex electrically evoqued in the context of cochlear implantation and fittings.

Even though the retrofacial approach can be used for other indication, the goal of the approach in this study is original.

I would like to discuss a few points with the authors in order to rise the interest of the paper:

- Introduction: ok

- Material and methods

1) How long did the segmentation and the surgical planning take to perform?

2) You describe an optimal head orientation, how could reach the correct orientation in the operating room?

3) Wich facial nerve monitoring did you use?

4) What was the additional duration added to the surgery once the learning curve of stapedius muscle exposure was achieved?

- Results

1) You extracted many interesting metrics from the surgical planning tool (table 2 and 3)? Could you compare these simulated metrics with real intra operative measurements or measurements on postoperative ct scans?

2) Did you test the eSRT in late postoperative and was it preserved? (Did exposure of the body muscle induced fibrosis that could impair the reflex long term preservation?)

- Discussion :

1) The main point of the article that is not discussed is why the electrical measurement of eSRT would be better than a visual assessment such as describe by Weiss et al (Weiss BG, Söchting F, Bertlich M, Busch M, Blum J, Ihler F, Canis M. An Objective Method to Determine the Electrically Evoked Stapedius Reflex Threshold During Cochlea Implantation. Otol Neurotol. 2018 Jan;39(1):e5-e11.)

Considering the risk for the facial nerve in non expert surgeons, would a video assessment yield to the same information? Was is the added value of an electrical measurement? The authors need to justify this comment to understand the goal of their study.

2) You tested this new approach in adults only in this study. Do you expect more difficulties to perform such an approach in pediatric cases?

3) Would you consider the use of a 30° endoscope to better expose the stapedius muscle body through an anterior approach?

-Conclusion :ok

-Figures : ok

Reviewer #3: This article presents a possible new approach to assessing the likely maximal stimulation levels that will be possible for people who will receive a cochlear implant. Cochlear implants enable otherwise profoundly deaf people to hear, they are typically very successful. Unlike many implants they are used across the lifespan with infants (18mo or less) through to older adults (90+ years) being eligible and gaining good hearing benefit. As more people, globally, receive implants there is a need to improve and enhance aspects of the assessment of the implants. Loudness of sounds delivered by an implant can be increased by changing the current release at the electrodes which are deep within the temporal bone in the cochlea. A limit to what can be achieved regarding loudness is affected by the point at which the amount of current being released in the cochlea starts to stimulate other major nerves including the facial nerve, due to unwanted current spread. The current assessment of the maximum stimulation is done by subjective reporting by the patient - in response to programming changes of the implant in the clinic. This is therefore hard to reliably evaluate in young children, people of all ages with complex needs and adults who may have cognitive decline. An alternative objective measurement would be valuable in improving patient outcomes. A single intraoperative test may also reduce follow up clinic time enabling greater capacity in clinics and lower burden on follow-ups for patients.

This paper builds on findings of previous work from the authors where surgical imaging and access to the stapedial muscle in a study of temporal bones enabled the identification of anatomical landmarks to support the choice of surgical access route to expose the belly of the stapedial muscle. This paper reports on reliable access to the belly of the muscle in a small cohort of people undergoing cochlear implantation. The value of this is that once accessed it may be possible to reliably measure the stimulation thresholds of this muscle, in-situ, during the surgical work-up for implantation. This would give objective data that could be used to predict the maximal likely tolerable stimulation levels that can be programmed into the cochlear implant, reducing the likelihood of discomfort and possible device under-use by patients. The paper also highlights the potential value of of using 3D reconstructive imaging during surgery, using a protocol that has not yet been fully validated - but that could improve outcomes if an algorithm can be developed from the landmark information recorded from the imaging.

The study is small - and the group sizes for the surgical approaches are therefore very small, this makes the statistical analysis indicative rather than absolute (hence the no for statistical reliability question). Based on the pilot data include a power calculation to determine the minimum group sizes likely to be needed for reliable analysis and data.

The approach reports reliable exposure of the belly of the stapedial muscle - however there is no report of the electrical stimulation or measurement of the stapedial responses. At a minimum the authors should consider describing the approach that will be used to make the measurement and to compare them with the current measures.

The patient cohort is not well described, basic audiological and outcome with the cochlear implant data for the cohort should be reported and compared with an appropriate control group that did not include exposure of the SM in the surgical protocol to ensure that outcomes are with the expected range despite the additional/alternative surgical approach.

Finally children are one of the likely groups where this may be of large benefit, while the direct lack of inclusion of children in the study is appropriate from an ethical point of view - comment on the imaging methods needed for the 3D reconstruction (X-ray doses) and the likelihood of being able to apply the landmark algorithm (once developed) in this population.

**********

6. PLOS authors have the option to publish the peer review history of their article (what does this mean?). If published, this will include your full peer review and any attached files.

If you choose “no”, your identity will remain anonymous but your review may still be made public.

**Do you want your identity to be public for this peer review?** For information about this choice, including consent withdrawal, please see our Privacy Policy.

Reviewer #1: No

Reviewer #2: No

Reviewer #3: No

**********

[NOTE: If reviewer comments were submitted as an attachment file, they will be attached to this email and accessible via the submission site. Please log into your account, locate the manuscript record, and check for the action link "View Attachments". If this link does not appear, there are no attachment files.]

While revising your submission, please upload your figure files to the Preflight Analysis and Conversion Engine (PACE) digital diagnostic tool, https://pacev2.apexcovantage.com/. PACE helps ensure that figures meet PLOS requirements. To use PACE, you must first register as a user. Registration is free. Then, login and navigate to the UPLOAD tab, where you will find detailed instructions on how to use the tool. If you encounter any issues or have any questions when using PACE, please email PLOS at figures@plos.org. Please note that Supporting Information files do not need this step.

# Author comment: Author response to Decision Letter 0

**DOI:** 10.1371/journal.pone.0272943.r002

21 Jul 2022

Point-by-point response to Reviewers

PONE-D-22-13880: Accessing the stapedius muscle via novel surgical retrofacial approach during cochlear implantation surgery: intraoperative results on feasibility and safety

We thank the reviewers for the detailed reviews and very helpful comments. We answer here all queries point-by point.

Reviewer #1

The authors investigated and compared the feasibility, reproducibility, and safety of two surgical approaches in cochlear implant surgery. The main aim of the study is to evaluate the stapedius muscle (SM) more objectively, via direct measurement from the muscle itself. With this hypothesis pre and retrofacial routes have been studied. Additionally, an image processing-based surgical planning tool in pre-surgical assessment was also investigated. Virtual 3D reconstruction of the temporal bone has been used lately especially in the robotic cochlear implantation and also for resident training programs.

The article, in general, is well written with very few grammatical errors.

Answer 1.1: The original draft was revised by a native speaker. The same native speaker checked the revised version.

In the methodology, the authors should specify the inclusion and exclusion criteria of the participants.

Answer 1.2: Thanks for this comment. We added the relevant inclusion criteria in the Methods on page 5: “The main inclusion criteria were as follows: (1) age ≥ 18 years; (2) need of a cochlear implantation; (3) measurable acoustical stimulation of the SR from the contralateral side.” There were no relevant exclusion criteria.

The main point of the discussion should focus on the benefit of SRT measurements directly form SM. The authors did not clarify this issue. Are there any investigations comparing the outcomes of traditional SRT measurements with the direct SM SRT measurements on the CI fittings and tuning?

Answer 1.3: This is a crucial issue, thanks. Reviewer #2 also raises this issue, therefore, please see also our answer 2.7. We added a completely new paragraph in the Discussion on page 17 giving an outlook what we plan next. All the investigations, the reviewer ask for, are not done yet. Reviewer #3 also gives a comment in this direction, please see answer 3.2.

Although retrofacial approach can be used in selected CI cases, the rationale on choosing this approach should be more clarified. Maybe a preliminary evaluation on the benefits of SRT measurements directly from SM will make this study more understandable and clear.

Answer 1.4: Yes, see 1.3. We emphasize more now the benefits of a SRT measurements directly from SM.

Reviewer #2

This study report the safety of a new indication of the retrofacial approach to expose the stapedius muscle body. The goal of the study is to allow the stapedius reflex electrically evoqued in the context of cochlear implantation and fittings. Even though the retrofacial approach can be used for other indication, the goal of the approach in this study is original. I would like to discuss a few points with the authors in order to rise the interest of the paper:

- Introduction: ok

- Material and methods

2.1 How long did the segmentation and the surgical planning take to perform?

Answer 2.1: We added information on the planning time on page 7: “The segmentation of one dataset took on average two hours.” In the next paragraph, we added in addition: “The surgical planning tool took on average 30 min to process one dataset and output the results.”

2) You describe an optimal head orientation, how could reach the correct orientation in the operating room?

Answer 2.2: We added a sentence on page 7: “For optimal head orientation, the patient’s head was rotated until the intraoperative situs matched to the image of the planning tool.”

3) Which facial nerve monitoring did you use?

Answer 2.3: We added this information on page 9: “Standard two-channel facial nerve monitoring was used.”

4) What was the additional duration added to the surgery once the learning curve of stapedius muscle exposure was achieved?

Answer 2.4: Many. Again important point. We added on page 9: “Once the learning curve of the SM exposure was achieved, the additional duration added to the standard CI surgery was 20 to 30 min.”

Results

1) You extracted many interesting metrics from the surgical planning tool (table 2 and 3)? Could you compare these simulated metrics with real intra operative measurements or measurements on postoperative ct scans?

Answer 2.5: No, we did not make intraoperative real time measurements or measurements on postoperative CT scans. 

2) Did you test the eSRT in late postoperative and was it preserved? (Did exposure of the body muscle induced fibrosis that could impair the reflex long term preservation?)

Answer 2.6: Another very important issue. We added in the Methods on page 10 the important information: “The follow-up of the patients within the study ended on average 4-5 days after surgery with the discharge from the hospital”. This means, that we do not know yet if the exposure induces fibrosis. We had to check the charts again for the longer follow-up. Reflex data after completion of the wound healing we have only for 4 patients, hence this not serious to report. Furhtermore, we added in the Results on page 12: “At the end of the surgery, the SR, stimulated on the contralateral side and measured on implantation side, disappeared but recovered during follow-up 6 weeks later.” We added in the Discussion on page 18: “Moreover, a longer follow-up period is needed to evaluate if the exposure of the SM induces fibrosis that could impair the SR long term preservation.”

In addition, we added one sentence at the end of the Results on page 12: “One patient had a transient postoperative facial palsy that recovered with one month. No other complications were seen.” The additional part of the surgery did not only include the exposure of the muscle but also a larger exposure of the facial nerve when preparing the retrofacial approach.

Discussion :

1) The main point of the article that is not discussed is why the electrical measurement of eSRT would be better than a visual assessment such as describe by Weiss et al (Weiss BG, Söchting F, Bertlich M, Busch M, Blum J, Ihler F, Canis M. An Objective Method to Determine the Electrically Evoked Stapedius Reflex Threshold During Cochlea Implantation. Otol Neurotol. 2018 Jan;39(1):e5-e11.) Considering the risk for the facial nerve in non expert surgeons, would a video assessment yield to the same information? Was is the added value of an electrical measurement? The authors need to justify this comment to understand the goal of their study.

Answer 2.7: Thanks for the reference to the publication of Weiss et al. We added this publication to the reference (reference #39). Reviewer #1 also addressed this issue, see also answer 1.3. Reviewer #3 also gives a comment in this direction, please see answer 3.2. We added a completely new paragraph in the Discussion on page 17: “The current standard for intraoperative eSRT measurements is the visual identification of the SR by the surgeon. Unfortunately, this method is highly subjective and hampered by ventilation-associated situs excursions, blinking, or other distractions [39]. Recently, Weiss et al. presented a tracking software to quantify stapes head movements automatically [39]. So far, the system was tested postoperatively against visual registration on the video material. We want to go one step further by recording of a needle EMG of the SM during the eSRT measurement as the absolute proof of an activation of the SR. Such recordings are in the next clinical trial. Furthermore, the ultimate goal is to permanently implant this recording electrode in the SM like implanting the CI in the cochlea. This would allow an objective and long-term eSRT fitting of the CI.”

2) You tested this new approach in adults only in this study. Do you expect more difficulties to perform such an approach in pediatric cases?

Answer 2.8: No, we do not expect more difficulties, but this needs to shown in future studies. Therefore, we added in the Discussion on page 16 at the end: “Furthermore, only adult patients were analyzed. Further trials have to verify that the approach is also feasible in children.”

3) Would you consider the use of a 30° endoscope to better expose the stapedius muscle body through an anterior approach?

Answer 2.9: This is a good idea (to be checked!) but might have not that much impact. As we write in the Discussion on page 15: The anterior part (that is seen or better seen by a 30° endoscope) contains primarily tendinous tissue. Of importance is the inferior compartment with the muscle tissue (better approached in most cases by the retrofacial access.

-Conclusion :ok

-Figures : ok

Reviewer #3

This article presents a possible new approach to assessing the likely maximal stimulation levels that will be possible for people who will receive a cochlear implant. Cochlear implants enable otherwise profoundly deaf people to hear, they are typically very successful. Unlike many implants they are used across the lifespan with infants (18mo or less) through to older adults (90+ years) being eligible and gaining good hearing benefit. As more people, globally, receive implants there is a need to improve and enhance aspects of the assessment of the implants. Loudness of sounds delivered by an implant can be increased by changing the current release at the electrodes which are deep within the temporal bone in the cochlea. A limit to what can be achieved regarding loudness is affected by the point at which the amount of current being released in the cochlea starts to stimulate other major nerves including the facial nerve, due to unwanted current spread. The current assessment of the maximum stimulation is done by subjective reporting by the patient - in response to programming changes of the implant in the clinic. This is therefore hard to reliably evaluate in young children, people of all ages with complex needs and adults who may have cognitive decline. An alternative objective measurement would be valuable in improving patient outcomes. A single intraoperative test may also reduce follow up clinic time enabling greater capacity in clinics and lower burden on follow-ups for patients.

This paper builds on findings of previous work from the authors where surgical imaging and access to the stapedial muscle in a study of temporal bones enabled the identification of anatomical landmarks to support the choice of surgical access route to expose the belly of the stapedial muscle. This paper reports on reliable access to the belly of the muscle in a small cohort of people undergoing cochlear implantation. The value of this is that once accessed it may be possible to reliably measure the stimulation thresholds of this muscle, in-situ, during the surgical work-up for implantation. This would give objective data that could be used to predict the maximal likely tolerable stimulation levels that can be programmed into the cochlear implant, reducing the likelihood of discomfort and possible device under-use by patients. The paper also highlights the potential value of of using 3D reconstructive imaging during surgery, using a protocol that has not yet been fully validated - but that could improve outcomes if an algorithm can be developed from the landmark information recorded from the imaging.

The study is small - and the group sizes for the surgical approaches are therefore very small, this makes the statistical analysis indicative rather than absolute (hence the no for statistical reliability question). Based on the pilot data include a power calculation to determine the minimum group sizes likely to be needed for reliable analysis and data.

Answer 3.1: Yes, this was the first step, a study on feasibility and safety (as we mention it already in the title). As we write in the Introduction: “The exposed SM provides a route for directly measuring electromyographic muscle activity (EMG) and, therefore, reliable intraoperative measurement of the eSRT”. Next step, we will record muscle potentials from the exposed SM. When we have first data from the EMG recordings, it is time to make a power analysis for a first prospective observational trial. At the moment, it seems to be too early for a power analysis. Furthermore, such a power analysis will be part of the Methods in the publication of the next study, but is normally not presented in a Discussion for a prior study.

The approach reports reliable exposure of the belly of the stapedial muscle - however there is no report of the electrical stimulation or measurement of the stapedial responses. At a minimum the authors should consider describing the approach that will be used to make the measurement and to compare them with the current measures.

Answer 3.2: The study on the EMG recordings from the SM during eSRT measurement is ongoing. This will be published when the recruitment is finished and the results are analyzed. Therefore, we give now an outlook on this next step in a completely new paragraph in the Discussion on page 17, see also answer 2.7.

The patient cohort is not well described, basic audiological and outcome with the cochlear implant data for the cohort should be reported and compared with an appropriate control group that did not include exposure of the SM in the surgical protocol to ensure that outcomes are with the expected range despite the additional/alternative surgical approach.

Answer 3.3: This was a proof-of-principle study on the feasibility and safety of the access to the stapedius muscle. Audiological outcome was no secondary outcome criterion of the study and the follow-up ended with discharge of the patient. Reviewer #2 asked also a question in this direction. As we explain in answer 2.6, no relevant complication occurred. We added this in the Results. Patient for standard cochlear implant surgery were selected. Due to the query of Reviewer #1, see answer 1.2, we added the few inclusion criteria. As added in the Result, we had 1 patients (out of 16 = 6.3%) with a transient facial palsy. This is in the normal range of this complication of CI surgery. To emphasize this, we added on page 17 at the beginning of the Discussion: “No major complications occurred. Especially, the rate of transient postoperative facial palsy was in the expected range and no permanent facial palsy occurred. ”

Finally children are one of the likely groups where this may be of large benefit, while the direct lack of inclusion of children in the study is appropriate from an ethical point of view - comment on the imaging methods needed for the 3D reconstruction (X-ray doses) and the likelihood of being able to apply the landmark algorithm (once developed) in this population.

Answer 3.4: Reviewer #2 also asked for the role of the access in children. As we stated in answer 2.8 and added this in the Discussion, we do not expect major differences in children but we do not know it. This was a first feasibility study. Ethical reasons drives us to start to evaluate the access in adults. Next, we will perform EMG recording from the SM in adults. If this reveals promising results, it is time to think about inclusion of children in further trials. Therefore, is seems to be too early the pros and cons for the use in children. All older children and CI candidates get a DYNA-CT of the temporal bone in the planning phase (as the adults in this study pre- and postop; actually, DYNA-CT data was used for the segmentation) The radiation exposure during DYNA-CT is much lower than with conventional CT or HRCT of the temporal bone. In the Discussion on page 18, we added the sentence: “In children, also the pros and cons of an additional 3D reconstruction based on CT data has to be weighed against the radiation exposure. In the present study, we used a low-dose DYNA-CT protocol.”

Orlando Guntinas-Lichius

for all authors

Jena, 20-July-2022

Attachment Submitted filename: Rebuttal v3.docx Click here for additional data file.

# Peer review: Decision Letter 1

**DOI:** 10.1371/journal.pone.0272943.r003

**Authors:** Adrien A. Eshraghi

1 Aug 2022

Accessing the stapedius muscle via novel surgical retrofacial approach during cochlear implantation surgery: intraoperative results on feasibility and safety

PONE-D-22-13880R1

Dear Dr. Guntinas-Lichius,

We’re pleased to inform you that your manuscript has been judged scientifically suitable for publication and will be formally accepted for publication once it meets all outstanding technical requirements.

Within one week, you’ll receive an e-mail detailing the required amendments. When these have been addressed, you’ll receive a formal acceptance letter and your manuscript will be scheduled for publication.

An invoice for payment will follow shortly after the formal acceptance. To ensure an efficient process, please log into Editorial Manager at http://www.editorialmanager.com/pone/, click the 'Update My Information' link at the top of the page, and double check that your user information is up-to-date. If you have any billing related questions, please contact our Author Billing department directly at authorbilling@plos.org.

If your institution or institutions have a press office, please notify them about your upcoming paper to help maximize its impact. If they’ll be preparing press materials, please inform our press team as soon as possible -- no later than 48 hours after receiving the formal acceptance. Your manuscript will remain under strict press embargo until 2 pm Eastern Time on the date of publication. For more information, please contact onepress@plos.org.

Kind regards,

Adrien A. Eshraghi, M.D.

Academic Editor

PLOS ONE

Additional Editor Comments (optional):

Reviewers' comments:

Reviewer's Responses to Questions

**Comments to the Author**

1. If the authors have adequately addressed your comments raised in a previous round of review and you feel that this manuscript is now acceptable for publication, you may indicate that here to bypass the “Comments to the Author” section, enter your conflict of interest statement in the “Confidential to Editor” section, and submit your "Accept" recommendation.

Reviewer #2: All comments have been addressed

Reviewer #3: All comments have been addressed

**********

2. Is the manuscript technically sound, and do the data support the conclusions?

The manuscript must describe a technically sound piece of scientific research with data that supports the conclusions. Experiments must have been conducted rigorously, with appropriate controls, replication, and sample sizes. The conclusions must be drawn appropriately based on the data presented. 

Reviewer #2: Yes

Reviewer #3: Yes

**********

3. Has the statistical analysis been performed appropriately and rigorously? 

Reviewer #2: Yes

Reviewer #3: N/A

**********

4. Have the authors made all data underlying the findings in their manuscript fully available?

The PLOS Data policy requires authors to make all data underlying the findings described in their manuscript fully available without restriction, with rare exception (please refer to the Data Availability Statement in the manuscript PDF file). The data should be provided as part of the manuscript or its supporting information, or deposited to a public repository. For example, in addition to summary statistics, the data points behind means, medians and variance measures should be available. If there are restrictions on publicly sharing data—e.g. participant privacy or use of data from a third party—those must be specified.

Reviewer #2: Yes

Reviewer #3: Yes

**********

5. Is the manuscript presented in an intelligible fashion and written in standard English?

PLOS ONE does not copyedit accepted manuscripts, so the language in submitted articles must be clear, correct, and unambiguous. Any typographical or grammatical errors should be corrected at revision, so please note any specific errors here.

Reviewer #2: Yes

Reviewer #3: Yes

**********

6. Review Comments to the Author

Please use the space provided to explain your answers to the questions above. You may also include additional comments for the author, including concerns about dual publication, research ethics, or publication ethics. (Please upload your review as an attachment if it exceeds 20,000 characters)

Reviewer #2: all comments have been adresssed.

Reviewer #3: The authors have addressed the issues raised and the manuscript has been improved with key points clarified.

**********

7. PLOS authors have the option to publish the peer review history of their article (what does this mean?). If published, this will include your full peer review and any attached files.

If you choose “no”, your identity will remain anonymous but your review may still be made public.

**Do you want your identity to be public for this peer review?** For information about this choice, including consent withdrawal, please see our Privacy Policy.

Reviewer #2: No

Reviewer #3: **Yes: **Tracey A Newman

**********

# Editor report: Acceptance letter

**DOI:** 10.1371/journal.pone.0272943.r004

**Authors:** Adrien A. Eshraghi

2 Aug 2022

PONE-D-22-13880R1 

Accessing the stapedius muscle via novel surgical retrofacial approach during cochlear implantation surgery: intraoperative results on feasibility and safety 

Dear Dr. Guntinas-Lichius:

I'm pleased to inform you that your manuscript has been deemed suitable for publication in PLOS ONE. Congratulations! Your manuscript is now with our production department. 

If your institution or institutions have a press office, please let them know about your upcoming paper now to help maximize its impact. If they'll be preparing press materials, please inform our press team within the next 48 hours. Your manuscript will remain under strict press embargo until 2 pm Eastern Time on the date of publication. For more information please contact onepress@plos.org.

If we can help with anything else, please email us at plosone@plos.org. 

Thank you for submitting your work to PLOS ONE and supporting open access. 

Kind regards, 

PLOS ONE Editorial Office Staff

on behalf of

Dr. Adrien A. Eshraghi 

Academic Editor

PLOS ONE
